# Research on sustainable collaborative scheduling problem of multi-stage mixed flow shop for crankshaft components

**DOI:** 10.1038/s41598-023-49519-x

**Published:** 2024-01-02

**Authors:** Liang Nie, Qinglei Zhang, Mengyu Feng, Jiyun Qin

**Affiliations:** 1 China Institute of FTZ Supply Chain, Shanghai Maritime University, Pudong, Shanghai, 201306 China; 2 Logistics Engineering College, Shanghai Maritime University, Pudong, Shanghai, 201306 China

**Keywords:** Mechanical engineering, Applied mathematics

## Abstract

The crankshaft manufacturing process primarily comprises machining, single jacket, and double jacket stages. These stages collectively produce substantial carbon emissions, which significantly impact the environment. Low-carbon energy development and humanity's future are closely related. To promote the sustainable development of crankshaft manufacturing enterprises and improve the production efficiency of crankshafts, research on sustainable collaborative scheduling problems in multi-stage mixed flow shop for crankshaft components is conducted. In addition, the transportation process of related workpieces in the crankshaft manufacturing process, which generally have a large mass, also produces substantial carbon emissions. This paper constructs a multi-objective integer optimization model based on the manufacturing process characteristics of crankshaft components, with minimizing the maximum manufacturing time and carbon emissions as optimization objectives. Considering the complexity of the problem, a comprehensive algorithm integrating moth-flame optimization and NSGA-III is used to solve the mathematical model. Through case experiments, the integrated algorithm is compared and analysed with four classic multi-objective optimization algorithms: NSGA-III, NSGA-II, MOEA/D, and MOPSO. The experiments demonstrate that the algorithm presented in this paper offers significantly enhanced optimization efficiency in solving the problem under study compared to other algorithms. Moreover, this paper compares multi-stage collaborative scheduling and non-collaborative scheduling in the crankshaft manufacturing process, ultimately demonstrating that collaborative scheduling is more conducive to the sustainable development of manufacturing enterprises. The results indicate that the annual carbon emissions can reduce about 3.6 ton.

## Introduction

In recent years, the rapid development of the manufacturing industry has greatly boosted China's economic growth. However, despite underpinning China's economic development, the manufacturing industry consumes a vast amount of resources and generates considerable pollution, leading to substantial environmental impact ^1^. Since the twenty-first century, China's manufacturing industry has accounted for 51% of the country's total energy consumption and 52% of total carbon emissions ^2^. Therefore, it is necessary to explore green manufacturing.

Zheng and Wang ^3^ studied the green scheduling problem of unrelated parallel machines with resource constraints and proposed a collaborative multi-objective fruit fly optimization algorithm to optimize the maximum manufacturing time and total carbon emissions. Wang et al. ^4^ studied the blocking flow shop scheduling problem to optimize its maximum completion time and energy consumption. Sang and Tan ^5^ designed an optimization method called SV-MA to solve the high-dimensional multi-objective green flexible job shop scheduling problem, aiming to improve the production capacity and processing quality while reducing energy consumption. Qin et al. ^6^ investigated the green job shop scheduling problem with variable processing speeds and designed a mixed-integer linear programming model and a knowledge-based multi-objective memory algorithm (MOMA) to solve the problem. Liu et al. ^7^ considered a more realistic dual flexible job shop scheduling problem and established a multi-objective mixed-integer programming model to optimize the maximum completion time, total worker cost, and the overall impact of green production indicators. Afsar et al. ^8^ considered the uncertainty of processing time and studied the workshop scheduling problem with the aim to minimize energy consumption during machine idle time and minimize the production cycle of projects.

Green scheduling is not just an effective means of realizing green manufacturing, but it also enables modern manufacturing enterprises to attain energy conservation and emission reduction. Previous studies have conducted extensive research on green scheduling problems, but relatively little consideration has been given to the transportation process during manufacturing. The transportation process of some large workpieces can also produce significant carbon emissions, and the transportation of workpieces can also affect the processing process.

Varela et al. ^9^ studied the collaborative manufacturing scheduling problem. Through an industrial case study, they proposed an operational scheduling system that integrates the proposed scheduling model and various types of solution algorithms. Sang and Tan ^10^ studied the multi-objective distributed flexible job shop collaborative scheduling problem in intelligent factories and constructed a distributed flexible job shop collaborative scheduling model that simultaneously optimizes economic and green indicators. Due to production or environmental reasons, most products need to go through multiple production workshops, from raw materials to finished products. Qin et al. ^11^ researched the optimization framework of serial multi-workshop collaborative scheduling problems, compared the optimization results of different frameworks, and analyzed the results of each optimization framework. Tian et al. ^12^ used the Analytical Target Cascading (ATC) method to solve the collaborative optimization problem of cutting parameters, process routes, and scheduling (CCPJS). Grabot et al. ^13^ studied the industrial scheduling problem of collaboration between humans and software, analyzed the specific requirements of collaborative methods, and proposed solutions for visualization tools, constraint management, and interactive planning. Moon et al. ^14^ proposed a method to perform independent scheduling at the network edge through collaborative scheduling between edge devices in a multi-access edge computing structure to solve the job shop scheduling problem.

Existing research on workshop scheduling often concentrates on a single workshop, assuming that all workpieces are prepared beforehand. However, in actual production, the commencement time of workpieces depends on the production of corresponding workpieces in the preceding workshop. Therefore, the collaborative scheduling of multiple workshops with process constraints can help achieve the optimization of overall scheduling effects. Compared with single workshop scheduling, the decision variables of multi-workshop collaborative scheduling optimization in the crankshaft manufacturing process in this paper have multiplied, significantly increasing the difficulty of solving.

Chen et al. ^15^ conducted research on capacity, operation sequence, and other constraints in multi-order environments and used a mixed-integer programming model to achieve advanced planning and scheduling. Liu et al. ^16^ studied the workshop scheduling problem for low-volume, multi-variety manufacturing industries, and in order to obtain near-optimal solutions with quantifiable quality within strict time constraints, they proposed a new integer linear programming formulation to minimize the total weighted tardiness. Salido et al. ^17^ designed a multi-objective genetic algorithm to obtain a trade-off between maximum duration and energy consumption. Qin et al. ^18^ developed a multi-objective genetic algorithm to minimize total energy consumption and total weighted tardiness. Mahmud et al. ^19^ developed an improved multi-objective particle swarm algorithm for problems that can provide highly customized and on-time delivery requirements at the lowest cost, and experimentally demonstrated the superiority of the algorithm. Dou et al. ^20^ designed a multi-objective particle swarm optimization algorithm based on crowding distance and external Pareto archive for the integrated optimization problem of reconfigurable manufacturing system configuration design and scheduling, to find the compromise solution between total cost and tardiness. Li et al. ^21^ proposed a problem-specific heuristic improved decomposition-based multi-objective evolutionary algorithm for solving the batch flow problem of Hybrid Flow Shop Scheduling (HFSP). Sun et al. ^22^ considered the flexible job shop scheduling with uncertain processing times represented by fuzzy numbers and proposed an effective hybrid cooperative evolutionary algorithm (hCEA) to minimize the fuzzy maximum completion time. Stastny et al. ^23^ introduced a new graph-based algorithm draft for optimizing scheduling problems.

Scheduling problem-solving algorithms include two categories: exact algorithms and heuristic algorithms. While exact algorithms can yield more accurate solutions, they are inefficient and are only applicable to small-scale scheduling problems. On the other hand, intelligent approaches can effectively tackle large-scale scheduling issues. In recent times, scholars have widely utilized intelligent optimization algorithms ^24–29^.

The previous studies extensively explore green scheduling and collaborative manufacturing, focusing on optimizing manufacturing time, carbon emissions, energy consumption, and total worker cost. Various models and algorithms, such as the multi-objective fruit fly optimization, mixed-integer linear programming, and multi-objective genetic algorithm, are proposed and tested. However, the underemphasis on the transportation process during manufacturing is lack of study. Also, the contribution of the transportation process to carbon emissions and more efficient algorithms to solve large-scale scheduling problems are still needed to be addressed.

Optimizing workshop scheduling can enhance production efficiency, minimize resource waste and environmental pollution, thereby reducing costs and boosting corporate competitiveness. This aligns with national policy requirements and supports sustainable development. The effective strategy of reducing carbon emissions lies in decreasing pollutant generation and energy consumption via parameter optimization during the manufacturing process. This study concentrates on the collaborative scheduling scheme optimization for multi-stage flexible manufacturing workshops in the crankshaft manufacturing process. Past studies on scheduling optimization primarily focused on machining workshops, overlooking the significant carbon emissions and manufacturing time consumed during the single jacket and double jacket stages. These stages are characterized by distinct manufacturing processes, with processing sequence constraints for some components in the double jacket stage significantly impacting overall manufacturing time. Additionally, this study considers the transportation process of workpieces among various stations and machines, using two overhead cranes and one transfer vehicle. The complexity of the study is amplified by the inclusion of the transportation process and the coordination of multiple transportation equipment.

This paper presents a unique approach to the sustainable optimization of scheduling for multi-stage mixed flow shops, emphasizing the efficient coordination of flexible mixed flow shops in crankshaft component manufacturing. The innovative aspect lies in aiming to minimize both the maximum manufacturing time and total carbon emissions throughout the process. Key considerations include carbon emissions from workpiece processing, machine idling, and transportation. The study also sheds light on the processing sequence of workpieces and the transportation paths used within the manufacturing environment, leading to optimized scheduling outcomes. The main contributions of this work are twofold.

1. It introduces a sustainable collaborative scheduling model specifically tailored to the complex manufacturing process of crankshafts. This model encompasses the processing of workpieces at multiple stages, and the inter-machine transportation process.
2. The paper proposes a modified NSGA-III algorithm incorporating the MFO method to solve the multi-objective optimization model, thereby enhancing its practical applicability.
3. The effectiveness of the proposed algorithm and model is demonstrated through a real-world case study, confirming the potential of this approach to significantly improve manufacturing efficiency and environmental impact.

The remaining content of this paper is organised as follows: “Introduction” summarizes and analyses the current research status in related fields. Section “Problem description and symbols” provides a more detailed description of the research problem and establishes a multi-objective optimization model for this problem. Section “Algorithm flow” introduces the process of the NSGA-III-MFO algorithm. Section “Case study” validates the mathematical model and optimization algorithm constructed in the previous two sections based on a specific crankshaft production case, proving the effectiveness of the model and the superiority of the intelligent algorithm. Section “Conclusions” concludes the research in this paper and introduces the prospects for future research.

## Problem description and symbols

**Figure 1:**
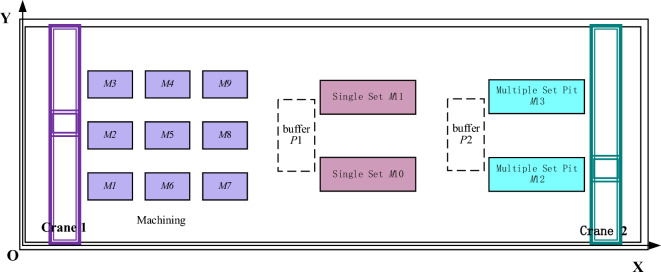
Layout diagram of crankshaft manufacturing workshop.

This paper studies the multi-stage hybrid flow shop sustainable collaborative scheduling problem in the crankshaft manufacturing process, which can be described as follows: assume there are M machine groups and n workpieces. Workpiece i can be processed through different deterministic processes. Each process encompasses one or more machines, k, with corresponding capabilities. As shown in Fig. 1, during the machining stage, workpieces are transported between various processing machines via overhead crane. (1) Upon completion of the last machining operation, the workpiece is transferred to buffer area 1 by the same crane. Overhead crane 2 transports the workpiece from buffer area 1 to the single-fit area for single-fit operations. After the single-fit operation is completed, the transfer vehicle transports the single-fit pieces to buffer area. (2) Overhead crane 2 is responsible for transporting workpieces from the two buffer areas to the re-fit pit for re-fit operations. Throughout the manufacturing process, there are precedence constraints on the processing operations of the workpieces. However, there are no order constraints between the same type of identical workpieces, such as no precedence constraint between the processing of two identical bends. This paper establishes a mathematical model for this problem, with the aim of minimizing the time consumed and total carbon emissions in the entire manufacturing process, the parameters of the model is given in Table 1.

**Table 1:** Symbol description.

Symbol	Description	Symbol	Description
*n*	Number of workpieces $\left(i\in 1, 2,\dots ,n\right)$	$p_{c}^{k}$	Processing power of machine k
*m*	Number of machining machines $\left(k\in 1, 2,\dots ,{m}^{\mathrm{^{\prime}}}\right)$ Single set induction heating device $\left(k\in {m}^{\mathrm{^{\prime}}}+1,\dots ,m\mathrm{^{\prime}}\mathrm{^{\prime}}\right)$ Multiple set induction heating device $\left(k\in m\mathrm{^{\prime}}\mathrm{^{\prime}}+1,\dots ,m\right)$	$p_{idle}^{k}$	Idle power of cutting machine k
${q}_{i}$	Total number of processes for workpiece $i$	$t_{c}^{k} (O_{ij} )$	Processing time of machine k
${O}_{ij}$	Process j of workpiece i($j\in [1,{q}_{i}]$)	$t_{idle}^{k} (O_{ij} )$	Waiting time of machine k
$M({O}_{ij})$	Machines available for process ${O}_{ij}$	*P*_*s*_	Idle power of overhead crane
$\eta_{e}$	Electricity carbon emission coefficient	*p*	Heating power of induction heating device
*tijk*	Working time of process ${O}_{ij}$ on machine k	${k}_{x}$	X-axis coordinate of machine k in the workshop
${a}_{ij}^{k}$	Processing decision variable for process ${O}_{ij}$(1 if processed on machine k, otherwise 0)	${k}_{y}$	Y-axis coordinate of machine k in the workshop
*Є1*	Energy consumption per unit distance for overhead crane (loaded)	${h}_{z}$	Lifting height of workpiece in the z-axis direction
*Є2*	Energy consumption per unit distance for trailer (loaded)	${v}_{g}$	Horizontal travel speed of overhead crane
*Є3*	Energy consumption per unit distance for overhead crane (empty)	${v}_{t}$	Vertical travel speed of overhead crane
*Є4*	Energy consumption per unit distance for trailer (empty)	${v}_{s}$	Lifting speed of overhead crane for workpieces
$\theta_{g}$	Efficiency of overhead crane in horizontal transportation	${v}_{d}$	Release speed of workpieces
$\theta_{t}$	Efficiency of overhead crane in vertical transportation	$v_{x}$	Transportation speed of trailers
$\theta_{s}$	Efficiency of overhead crane in lifting mechanism	$st_{ijk}$	Setup time for process ${O}_{ij}$ on machine k
$\theta_{a}$	Transportation efficiency of trailer	$s_{ig}^{k}$	1 if bend i and shaft diameter g are processed together on machine k, otherwise 0
$x_{a}$	Distance of transportation from single set area to multiple set area	*F*	Number of crankshafts($f \in (1,...,F)$)
$a_{ij}^{t}$	1 if process $O_{ij}$ uses trailer transportation, otherwise 0	${St}_{ij}^{k}$	Start time of process ${O}_{ij}$ on machine k
$b({O}_{i1j1},{O}_{ij})$	Empty transportation decision variable (1 if process ${O}_{ij}$ follows process ${O}_{{\text{i}}1j1}$ in process sequence, otherwise 0)	${Ft}_{ij}^{k}$	Completion time of process ${O}_{ij}$ on machine k
$c\left({k}_{i\left(j-1\right)},{{k}_{1}}_{ij}\right)$	Loaded transportation decision variable (1 if process ${O}_{i(j-1)}$ is processed on machine k and process ${O}_{ij}$ is processed on machine ${k}_{1}$, otherwise 0)	$d({k}_{i2j2},{k}_{ij})$	Adjacent process decision variable on machine (1 if process ${O}_{ij}$ follows process ${O}_{i2j2}$ on machine k, otherwise 0)

### Assumption

In order to ensure that the proposed model eliminates the interference of all irrelevant factors, the following assumptions are made.

1. All processing parameters of workpieces are determined, which means that the crankshaft machining process is constrained by the parameters of the processing equipment.
2. Each workpiece includes multiple processes, and each process can be executed on a machine with corresponding capabilities.
3. There are no priority constraints between different workpieces during the machining stage, but there are priority constraints between the operation sequences of the same workpiece.
4. Subsequent operations cannot start if the previous operation has not been completed.
5. Each machine processes only one workpiece at a time, and each workpiece can only be processed on one machine at a time.
6. Machines operate normally without interruption due to malfunction.
7. The first process of each workpiece does not require transportation.
8. During the single-fit process, it is necessary to ensure that the corresponding shaft diameter has been processed and transported to the single-fit area when the crank bend heating is completed.
9. There are temporary storage areas in the single-fit and re-fit areas.
10. To simplify the model, the process of using overhead cranes to lift electromagnetic induction heating devices during the single and re-fit process is ignored.
11. Different transport machines do not interfere with each other.

### Transportation time and carbon emission model

#### Transportation time functions

Considering the large area occupied by heavy-duty workpiece processing workshops and the long distances between machines, the startup and braking times of each mechanism of the overhead crane transportation equipment are ignored. The transportation time formulas for each mechanism are as follows:

Unloaded transportation time for operation ${O}_{ij}$:

1 $$ t^{nl} (O_{ij} ) = \left( {\frac{{|k_{x} - k_{{1_{x} }} |}}{{v_{g} }} + \frac{{|k_{y} - k_{{1_{y} }} |}}{{v_{t} }}} \right)*a_{i(j - 1)}^{k} *a_{i1ji}^{{k_{1} }} $$

Unloaded waiting time for operation ${O}_{ij}$:

2 $$ Wt_{nl} (O_{ij} ) = \max [(St_{i1j1}^{{k_{1} }} + t^{nl} (O_{ij} ) - Ft_{i(j - 1)}^{k} ),0] $$

Total loaded transportation time for operation ${O}_{ij}$:

3 $$ t^{l} (O_{ij} ) = \left( {\frac{{|k_{x} - k_{{2_{x} }} |}}{{v_{g} }} + \frac{{|k_{y} - k_{{2_{y} }} |}}{{v_{t} }} + \frac{{h_{z} }}{{v_{s} }} + \frac{{h_{z} }}{{v_{d} }}} \right)*a_{i(j - 1)}^{k} *a_{ij}^{{k_{2} }} $$

Loaded transportation waiting time:

4 $$ Wt_{l} (O_{ij} ) = \max [(Ft_{i2j2}^{{k_{2} }} - t^{l} (O_{ij} ) - Ft_{i(j - 1)}^{k} ),0] $$

Time to transport a single set from the single set area to the double set temporary storage area using a tow truck:

5 $$ t^{\prime} = \frac{{x_{a} }}{{v_{x} }} \times a_{ij}^{t} $$

Total transportation waiting time for operation ${O}_{ij}$::

6 $$ Wt(O_{ij} ){ = }Wt_{nl} (O_{ij} ) + Wt_{l} (O_{ij} ) $$

#### Carbon dioxide emissions

Based on the carbon emissions of each operation in production, the carbon emission calculation formula for different machines is given using the Intergovernmental Panel on Climate Change (IPCC) carbon emission accounting method. The IPCC is an abbreviation for the Intergovernmental Panel on Climate Change, which is a United Nations body for assessing science related to climate change. The IPCC reference model is the most widely used carbon emission calculation method. It originates from the report on the greenhouse effect issued by the United Nations Climate Change Committee, expressed as: Carbon Emission = Activity Data*Emission Factor.

1. Carbon emissions in machining process
  Machine tool machining energy consumption:
  7 $$ E_{c}  = \sum\limits_{{k = 1}}^{{m\prime }} {\sum\limits_{{i = 1}}^{n} {\sum\limits_{{j = 1}}^{{q_{i} }} {(p_{c}^{k} *t_{c}^{k} (O_{{ij}} )*a_{{ij}}^{k} )*\eta _{e} } } }  $$
  Total machine tool standby energy consumption:
  8 $$ E_{{idle}}  = \sum\limits_{{k = 1}}^{{m\prime }} {p_{{idle}}^{k} *t_{{idle}}^{k} *\eta _{e} }  $$
  Total energy consumption in the machining process:
  9 $$ E_{machine} = E_{c} + E_{idle} $$
2. Carbon emissions in single and double set processes
  Electromagnetic induction heating process carbon emissions:
  10 $$ E_{el} = \sum\limits_{i = 1}^{n} {\sum\limits_{j = 1}^{{q_{i} }} {\sum\limits_{k = m^{\prime} + 1}^{m} {pt_{ijk} \eta_{e} a_{ij}^{k} } } } $$
3. Carbon emissions in transportation process
  1. During the workpiece machining process, cranks and shaft diameters are transported from the machining area to the single set temporary storage area by Crane 1; free end and output end are transported to the single set temporary storage area by Crane 1 and then to the double set area by Crane 2. Carbon emissions from crane transportation, single set process, and double set process transportation are calculated. Crane 1 and Crane 2 have the same model.
    For operation ${O}_{ij}$:, crane loaded transportation energy consumption:
    11 $$ E^{l} (O_{ij} ) = \varepsilon_{1} \theta_{g} |k_{x} - k_{1x} |{ + }\varepsilon_{1} \theta_{t} |k_{y} - k_{1y} |{ + }\varepsilon_{1} \theta_{s} h_{z} $$
    Thus, crane loaded transportation carbon emissions:
    12 $$ E_{crane}^{l} = \sum\limits_{i = 1}^{n} {\sum\limits_{j = 2}^{{q_{i} }} {E^{l} (O_{ij} )} } *\eta_{e} $$
    For operation ${O}_{ij}$:, crane loaded waiting energy consumption:
    13 $$ WE^{l} (O_{ij} ) = Wt_{l} (O_{ij} )*P_{s} $$
    Thus, crane loaded waiting carbon emissions:
    14 $$ WE_{crane}^{l} = \sum\limits_{i = 1}^{n} {\sum\limits_{i2 = 1}^{n} {\sum\limits_{j = 2}^{{q_{i} }} {\sum\limits_{j2 = 1}^{{q_{i} }} {\sum\limits_{k = 1}^{m} {\sum\limits_{{k_{2} = 1}}^{m} {WE^{l} (O_{ij} )*c(k_{i(j - 1)} ,k_{{2_{ij} }} )*d(k_{{2_{i2j2} }} ,k_{{2_{ij} }} )*\eta_{e} } } } } } } $$
    Operation ${O}_{ij}$: crane unloaded transportation energy consumption:
    15 $$ E^{nl} (O_{ij} ) = \varepsilon_{3} \theta_{g} |k_{x} - k_{1x} |{ + }\varepsilon_{3} \theta_{t} |k_{y} - k_{1y} | $$
    Operation ${O}_{ij}$: crane unloaded waiting energy consumption:
    16 $$ WE^{nl} (O_{ij} ) = Wt_{nl} (O_{ij} )*P_{c} $$
    Therefore, the total carbon emissions for unloaded crane transportation:
    17 $$ E_{crane}^{nl} = \sum\limits_{i = 1}^{n} {\sum\limits_{i1 = 1}^{n} {\sum\limits_{j = 2}^{{q_{i} }} {\sum\limits_{j1 = 1}^{{q_{i} }} {E^{nl} (O_{ij} )*b(O_{i1j1} ,O_{ij} )*\eta_{e} } } } } $$
    Total carbon emissions for unloaded crane waiting transportation:
    18 $$ WE_{crane}^{nl} { = }\sum\limits_{i = 1}^{n} {\sum\limits_{i1 = 1}^{n} {\sum\limits_{j = 2}^{{q_{i} }} {\sum\limits_{j1 = 1}^{{q_{i} }} {WE^{nl} (O_{ij} )*b(O_{i1j1} ,O_{ij} )} } } } *\eta_{e} $$
    In summary, the total carbon emissions for this stage of crane transportation:
    19 $$ E_{crane} = E_{crane}^{l} + WE_{crane}^{l} + E_{crane}^{nl} + WE_{crane}^{nl} $$
  2. Single set transported from single set area to double set area using tow truck:
    20 $$ E_{a} = 2\sum\limits_{k = m^{\prime} + 1}^{m^{\prime\prime}} {\sum\limits_{i = 1}^{n} {\sum\limits_{j = 1}^{{q_{i} }} {(\varepsilon_{2} + \varepsilon_{4} )\theta_{a} x_{a} \times } } } a_{ij}^{k} $$
  3. Double set process
    The crane transports the single set from the double set buffer area to the double set pit, then returns to the double set buffer area with unloaded transportation. The carbon emissions for this process are as follows:
    21 $$ E_{b} = \sum\limits_{k = m^{\prime\prime} + 1}^{m} {\sum\limits_{i = 1}^{n} {\sum\limits_{j = 1}^{{q_{i} }} {[(\varepsilon_{1} + \varepsilon_{3} )(\theta_{s} h_{z} a_{ij}^{k} + \theta_{g} |k_{x} - k_{1x} | + \theta_{t} |k_{y} - k_{1y} |)]} } } $$

### Integrated optimization model

Manufacturing has always been the most important production goal. Therefore, we will optimize two objectives simultaneously, as shown in Eqs. (22) and (23), where T_min_ is the total manufacturing time, and E_min_ is the carbon emission. Carbon emissions consumed during workshop scheduling mainly consist of machining carbon emissions, single-fitting carbon emissions, and double-fitting carbon emissions. The processing time of a workpiece is composed of the operation time and adjustment time on its corresponding machine.

22 $$ E_{min} = min(E_{machine} + E_{el} + E_{crane} + E_{a} + E_{b} ) $$

23 $$ T_{\min } = \min \{ \max [(Ft_{ij}^{k - 1} + t_{ijk} + st_{ijk} )*a_{ij}^{k} + Wt(O_{ij} )]\} $$

Constraint conditions:

24 $${\text{St}}\left({O}_{i\left(j+1\right)}\right)-Ft\left({O}_{ij}\right)\ge 0;$$

25 $$\sum_{k=1}^{m}{a}_{ij}^{k}=1;$$

26 $${\text{St}}\left({k}_{i+1}\right)-Ft\left({k}_{i}\right)\ge 0;$$

27 $${St}_{ij}^{k}+{t}_{ijk}+{st}_{ijk}={Ft}_{ij}^{k};$$

28 $${P}_{nl}\left({O}_{i1}\right)={P}_{l}\left({O}_{i1}\right)={P}_{l}\left({O}_{i1j1}\right);$$

29 $${P}_{nl}\left({O}_{ij}\right)={P}_{l}\left({O}_{ij}\right)={P}_{l}\left({O}_{i1j1}\right);$$

30 $${\text{Ft}}\left({O}_{ij}\right)+{Wt}_{l}\left({O}_{ij}\right)\ge Ft\left({O}_{i2j2}\right);$$

31 $$ \sum\limits_{k = 1}^{m} {[Ft_{ij}^{k} - (St_{i(j - 1)}^{k} + t_{ijk} )] \times a_{ij}^{k} } \ge 0 $$

32 $$ Ft(O_{{iq_{i} }} ) \ge \sum\limits_{k = m^{\prime}}^{m^{\prime\prime}} {a_{ig}^{k} \times (Ft(O_{{g(q_{i} - 1)}} ) + Wt(O_{{g(q_{i} - 1)}} ) + t(O_{{g(q_{i} - 1)}} ))} $$

Equation (24) represents the workpiece operation sequence constraint, ${\text{St}}({O}_{i(j+1)})$ is the start time of operation ${O}_{i(j+1)}$, and $Ft({O}_{ij})$ is the end time of operation ${O}_{i(j+1)}$; Eq. (25) indicates that at the same time, one operation can only be processed by one machine; Eq. (26) states that at the same time, one machine can only process one operation, where ${\text{St}}({k}_{i+1})$ represents the start time of the (i + 1)th process on machine k, and $Ft({k}_{i})$ represents the end time of the ith process on machine k; Eq. (27) shows that the processing start of the workpiece is not allowed to be interrupted; Eq. (28) states that the first operation of the workpiece does not require crane transportation; Eq. (29) indicates that if the previous and subsequent operations of the same workpiece are processed on the same machine, no transportation is needed; Eq. (30) specifies that the start time of load transportation must begin after the destination machine is free. Constraint (31) refers to the priority relationship of all operations for workpiece i. Constraint (32) indicates that before the crank heating is completed, the corresponding shaft diameter machining is finished and transported to the single-fitting area.

## Algorithm flow

**Figure 2:**
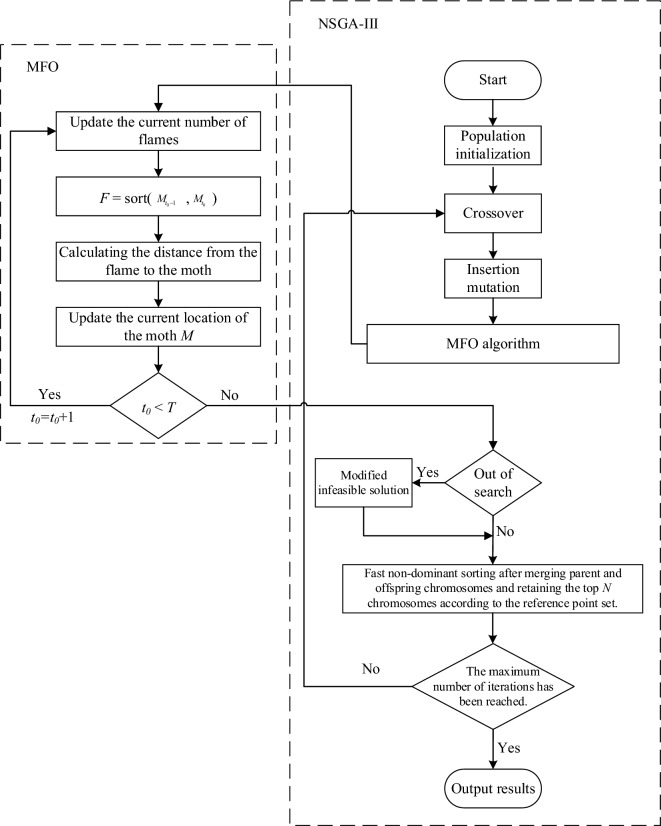
Algorithm flowchart.

This section proposes an intelligent optimization algorithm based on the improved NSGA-III algorithm using MFO to optimize the research problem. Figure 2 shows the algorithm flow of the optimization algorithm. Then, the encoding and decoding methods, as well as the specific implementation of NSGA-III and MFO, are introduced.

### Encoding

The encoding designed in this paper includes the processing sequence of workpieces in the machining workshop and the corresponding processing machines of the machining steps. Suppose a crankshaft needs to be machined, including two curved sections, two necks, one output end, and one free end. The machining steps for each workpiece and the corresponding machining time on each machine are shown in Table 2. The "-" indicates that the machining step cannot be processed on that machine. Workpiece has two identical workpieces $N_{1} ,N_{2}$, each with four steps. $N_{{3}} ,N_{{4}}$ are two identical workpieces, each with four steps. $N_{{5}}$, $N_{6}$ have three steps.

**Table 2:** Processing information.

Workpiece	Process	Machine processing time (min)					
		${M}_{1}$	${M}_{2}$	${M}_{3}$	${M}_{4}$	${M}_{5}$	${M}_{6}$
${N}_{1},{N}_{2}$	${O}_{11}$	2	4	–	–	–	–
	${O}_{12}$	4	1	3	–	–	–
	${O}_{13}$	–	–	–	4	5	–
	${O}_{14}$	–	–	–	–	–	6
${N}_{3},{N}_{4}$	${O}_{31}$	4	–	5	–	–	–
	${O}_{32}$	–	7	–	–	–	–
	${O}_{33}$	–	–	–	1	3	–
	${O}_{34}$	–	–	–	–	–	5
${N}_{5}$	${O}_{51}$	4	–	6	–	–	–
	${O}_{52}$	–	2	3	–	–	–
	${O}_{53}$	–	–	–	–	–	4
${N}_{6}$	${O}_{61}$	–	6	4	–	–	–
	${O}_{62}$	5	–	3	–	–	–
	${O}_{63}$	–	–	–	–	–	1

**Figure 3:**
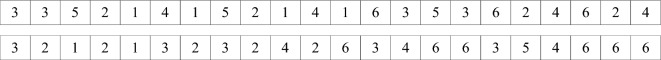
Encoding example diagram.

The encoding format of the scheduling task solution is shown in Fig. 3. The first row of numbers in Fig. 3 represents the workpiece number, and the appearance of the workpiece number represents the first machining step of the workpiece. The second appearance represents the second machining step. For example, in Fig. 3, the first machining step of workpiece 3 is performed first, then the second machining step of workpiece 3, followed by the first machining step of workpiece 5. The second row of numbers represents the machine number for the corresponding process, such as the first machining step of workpiece 3 being processed on machine 3 and the second machining step on machine 2.

### NSGA-III

#### Crossover

**Figure 4:**
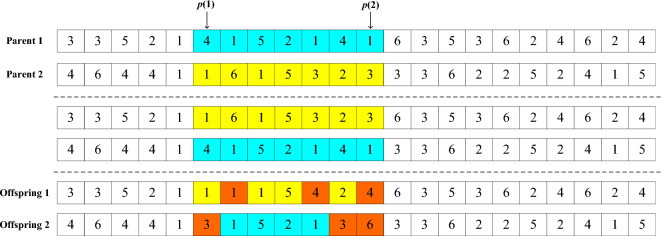
Crossover diagram.

This paper uses a two-point crossover method to generate offspring individuals. As shown in Fig. 3, two points, $p(1)$, $p(2)$, are randomly generated within the length range of the parent encoding. The segments between the two points of parent 1 and parent 2 are exchanged, resulting in the encoding in the middle of the two dashed lines in Fig. 4. At this point, the newly generated encoding may be unreasonable, as shown in the figure, with some repetition and omissions. After adjustment, the offspring 1 and offspring 2 encoding can be obtained, as shown at the bottom of Fig. 4.

#### Mutation

**Figure 5:**
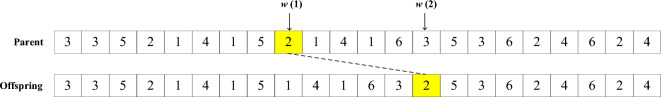
Mutation diagram.

In this study, the insertion method is used for performing mutation operations on the encoding. Firstly, two points, $w(1)$ and $w(2)$, are randomly generated within the range of encoding length, as illustrated in Fig. 5. The encoding at position $w(1)$ is inserted into position $w(2)$ to create a new offspring individual.

#### Elite selection strategy based on reference point set

To avoid the loss of superior individuals, the offspring population is merged with the parent population after being generated. The top NP chromosomes (assuming the population size is NP) are retained through Pareto non-dominated sorting. Individuals with smaller non-dominated ranks are prioritized to join the new generation St + 1. Most of the time, the number of individuals will exceed NP, while the number of individuals will be less than NP. In this case, an elite strategy based on reference point set is needed to select individuals from Fc + 1.

**Figure 6:**
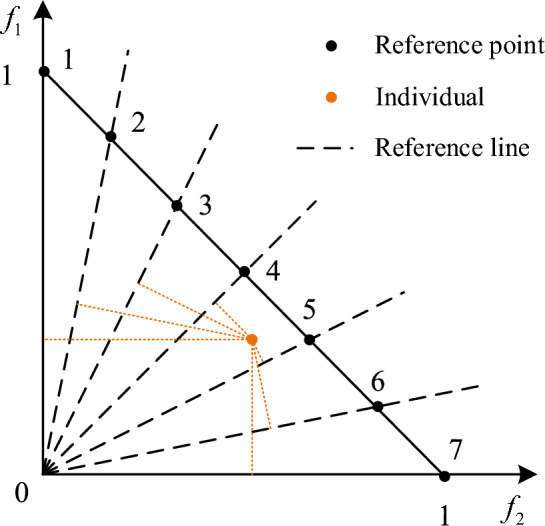
Illustration of correlation between population individuals and reference points.

First, a reference point set needs to be generated. Connect points (0, 1) and (1, 0). Divide the connecting line into several parts using the equal partition method, and the obtained points are the reference points. The lines connecting the origin to the reference points are the reference lines. Then, normalize the two objective values using formula (33) and map the normalized objective function values to the two-dimensional plane. Here, represents the minimum value of each objective in the current population. represents the intercept of the line connecting the extreme points of each objective direction with the coordinate axis. The calculation of the extreme points is shown in formula (34). As shown in Fig. 6, the individual is closest to the reference line of reference point 5, indicating that the individual is related to reference point 5.

33 $$ f_{i}^{n} ({\rm X}) = \frac{{f_{i} ({\rm X}) - z_{i}^{\min } }}{{a_{i} - z_{i}^{\min } }}\quad {\text{for }}i = 1,2 $$

34 $$ ASF(x,w) = \mathop {\max }\limits_{i = 1}^{2} (f_{i} ({\rm X}) - z_{i}^{\min } )/w_{i} \quad w_{i} = (\varepsilon , \ldots ,\varepsilon )^{T} ;\varepsilon = 10^{ - 6} ;w_{i}^{i} = 1 $$

Ultimately, the steps of the elite selection strategy based on the reference point set are as follows.

1. Count the number of individuals q_b_ related to each reference point.
2. Select the reference point b corresponding to the smallest number of individuals.
3. Check if the current set F_c+1_ contains any individuals related to reference point b. If it does, randomly select one of the related individuals to be a member of the offspring population, and $q_{b} = q_{b} + 1$. Otherwise, remove reference point b from the reference point set.
4. Repeat steps (2) and (3) until the number of individuals in the offspring population reaches NP.

### MFO

The inspiration for the Moth-Flame Optimization Algorithm comes from the navigation mechanism of moths. When moths fly at night, they usually guide their flight direction by maintaining the same angle with the moon. Since the distance between the Earth and the moon is very far, this method is feasible. However, in practice, moths can be deceived by artificial light sources. Due to the close distance between moths and artificial light sources, their navigation mechanism leads them to always spiral closer to the light source ^30^. The Moth-Flame Optimization Algorithm is designed based on the behavior of moths spiraling towards artificial light sources.

35 $$ M_{i} = S(M_{i} ,F_{j} ) $$

36 $$ S(M_{i} ,F_{j} ) = D_{i} *e^{bt} *\cos (2\pi t) + F_{j} $$

Equation (35) represents the position of each moth relative to the flame, where $M_{i}$ denotes the i-th moth, $F_{j}$ represents the j-th flame, and S is the spiral function. The flight path of moths can be simulated through Eq. (36), where $D_{i}$ denotes the distance from the jth flame to the ith moth, b is a constant defining the logarithmic spiral shape, and t is a random number in the range [− 1, 1] determining the subsequent distance from the moth to the flame. The smaller the t, the closer the moth is to the flame. Equation (37) calculates the distance D. The moths move in a spiral pattern around the flame, allowing the algorithm to explore the search space more thoroughly.

37 $$ D_{i} = \left| {F_{j} - M_{i} } \right| $$

38 $$ N_{{0}} = round\left( {N - l*\frac{N - 1}{T}} \right) $$

During the iterative process, to improve search efficiency, Eq. (38) provides an adaptive mechanism for the algorithm, where N represents the maximum number of flames, N0 represents the current number of flames, and T represents the maximum number of iterations. This adaptive mechanism enables the number of flames to decrease gradually with each iteration, retaining only the more optimal flames.

**Figure:**
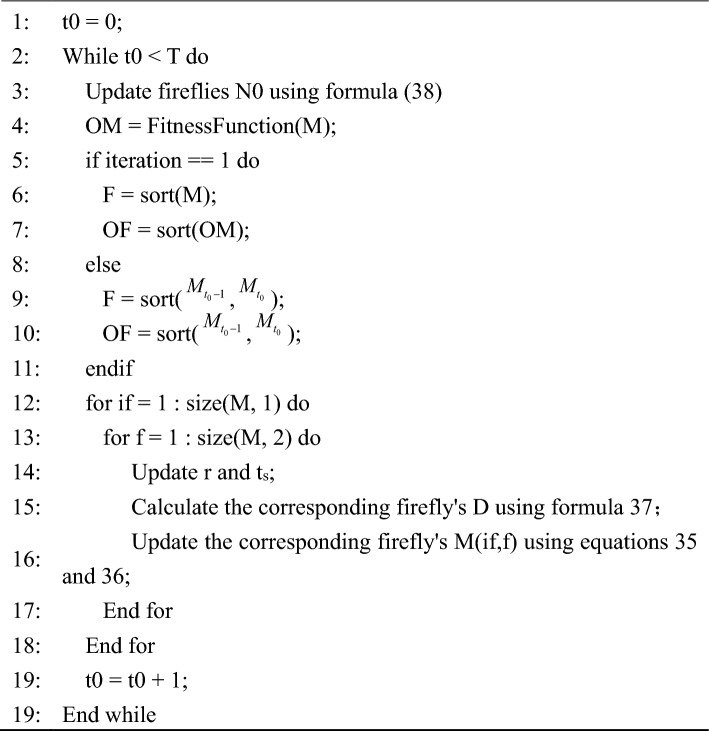
Algorithm MFO.

In this study, a multi-objective optimization model is established to minimize both the maximum manufacturing time and the total carbon emissions. Given that these two optimization objectives exhibit a nonlinear relationship, the paper employs a method to compute the Pareto optimal solution set. Pareto optimality refers to an ideal state of resource allocation where any change cannot improve at least one sub-objective without making any other sub-objective worse.

## Case study

### Experimental data

This article mainly studies the scheduling problem of machining, single set, and multi-set processes for crankshaft components. The case study in present study is derived from the crankshaft production workshop of Shanghai Marine Crankshaft Co., Ltd. Now, there is a need to produce two types of crankshafts, A and B. Crankshaft A contains nine bends, nine shaft diameters, one output end, and one free end. Crankshaft B contains six bends, six shaft diameters, one output end, and one free end. The gantry crane's horizontal transport speed is 40 m/min, and the vertical transport speed is 5 m/min. The transfer cart's unloaded transport power is 3 kW, the loaded power is 10 kW, and the transport speed is 30 m/min. The gantry crane's standby power is 5 kW, horizontal running power is 20 kW, vertical running power is 30 kW, lifting equipment weight is 6000 kg, and the crane weight is 10,000 kg. The electric energy carbon emission coefficient is 0.6752 kgCO_2_/Kw h ^31^. Other relevant workshop information is described in Table 3.

**Table 3:** Workshop information.

Equipment number	Name	Device name	Rated power (Kw)	Idle power (Kw)	Location (m)
${M}_{1}{,M}_{2}$	Machining	Bent vertical lathe	150	8	(20, 30), (20, 90)
${M}_{3}, {M}_{4}$	Machining	6.3-m vertical lathe	110	4	(20, 150), (100, 150)
${M}_{5}{, M}_{6}$	Machining	CNC boring and milling machine	55	3	(100, 90), (100, 30)
$${M}_{7}{, M}_{8}, {M}_{9}$$	Machining	CNC horizontal lathe	98	5	(180, 30), (180, 90)$$, $$(180, 150)
$${M}_{10}, {M}_{11}$$	Single set position	Induction heating device	30	1	(260, 50)$$, $$(260, 100)
$$M_{12}$$$$M_{13}$$	Multiple set pit	Induction heating device	30	1	(360, 50)$$, $$(360, 100)
${P}_{1}$	Single set temporary storage area	–	–	–	(220, 90)
${P}_{2}$	Multiple set temporary storage area	–	–	–	(310, 90)

The information pertaining to the workpieces used in the machining process of the two crankshafts is presented in Table 4. The processing of the workpiece encompasses both preparation and working times. It is important to highlight that after undergoing the single set process, the original two workpieces are integrated into a new entity, referred to as the "single set piece". Subsequent multi-set processes are then conducted on this single set piece. Consequently, in Table 4, the processing time for the multi-set process is only annotated subsequent to the bending entry, indicating that the bending and shaft neck do not undergo separate multi-set processes.

**Table 4:** Workpiece information.

Workpiece	Process	Setup time/operation time					
		${M}_{1},{M}_{2}$	${M}_{3},{M}_{4}$	${M}_{5}{,M}_{6}$	${M}_{7}{,M}_{8}{,M}_{9}$	${M}_{10},{M}_{11}$	${M}_{12},{M}_{13}$
A Bend ${N}_{1}$	${O}_{11}$	1/22				–	–
	${O}_{12}$	1/18				–	–
	${O}_{13}$			1/14		–	–
	${O}_{14}$		1/16.5			–	–
	${O}_{15}$			1/4		–	–
	${O}_{16}$					2/19	
	${O}_{17}$						2/24
A Shaft diameter ${N}_{2}$	${O}_{21}$				1/4		
	${O}_{22}$				1/4		
	${O}_{23}$				1/6		
	${O}_{24}$					1/2	
A Output end ${N}_{3}$	${O}_{31}$				1/5		
	${O}_{32}$				1/15		
	${O}_{33}$				1/3		
	${O}_{34}$						2/24
A Free end ${N}_{4}$	${O}_{41}$				1/5		
	${O}_{42}$				1/9		
	${O}_{43}$				1/3		
	${O}_{44}$						1/3
B Bend ${N}_{5}$	${O}_{51}$	1/22.5					
	${O}_{52}$	1/18					
	${O}_{53}$			1/12			
	${O}_{54}$		1/15.5				
	${O}_{55}$			1/3			
	${O}_{56}$					2/13	
	${O}_{57}$						2/15
B Main spindle neck ${N}_{6}$	${O}_{61}$				1/3.5		
	${O}_{62}$				1/4		
	${O}_{63}$				1/6		
	${O}_{64}$					1/2	
B Output end ${N}_{7}$	${O}_{71}$				1/5		
	${O}_{72}$				1/15		
	${O}_{73}$				1/3		
	${O}_{74}$						2/15
B Free end ${N}_{8}$	${O}_{81}$				1/4.5		
	${O}_{82}$				1/10		
	${O}_{83}$				1/3		
	${O}_{84}$						1/2

### Results and discussions

#### Algorithm parameter settings

In this section, the algorithm parameters are determined through experiments and by referring to previous research. Based on the scale of the case in this paper, the number of iterations is set to 500. Then, a preliminary experiment is conducted to determine the population size and mutation probability. The population size is set at three levels: 20, 30, and 50, while the mutation probability is set at two levels: 0.6 and 0.2. Since the Pareto front is not sensitive to changes in crossover probability, it is set to 0.6 based on experience.

For the case study, the algorithm is run 10 times sequentially to obtain the non-dominated solution set Pa (a = 1, 2, …, 18). By fixing one parameter, the Pa at different levels of the other parameter for each case is merged to obtain the FE. As shown in Table 5, the probabilities obtained from Pa for each FE are calculated as the result response values. According to Table 5a, regardless of whether the mutation probability is fixed at 0.6 or 0.2, the result response value is the highest when the population size is 50, so the population size is set to 50. When the population size is 50, the result response value is greater with a mutation probability of 0.2, so the mutation probability is set to 0.2.

**Table 5:** Parameter response values.

(a) Fixed mutation probability			(b) Fixed population size			
Population size	Mutation probability		Population size	Mutation Probability		总计
	0.6	0.2		0.6	0.2	
50	**46.79%**	**41.29%**	50	36.01%	**63.99%**	100%
30	20.91%	26.83%	30	**56.78%**	43.22%	100%
20	30.3%	31.89%	20	27.16%	**72.84%**	100%
Total	100.00%	100.00%				

Significant values are in bold.

#### Algorithm performance analysis

**Figure 7:**
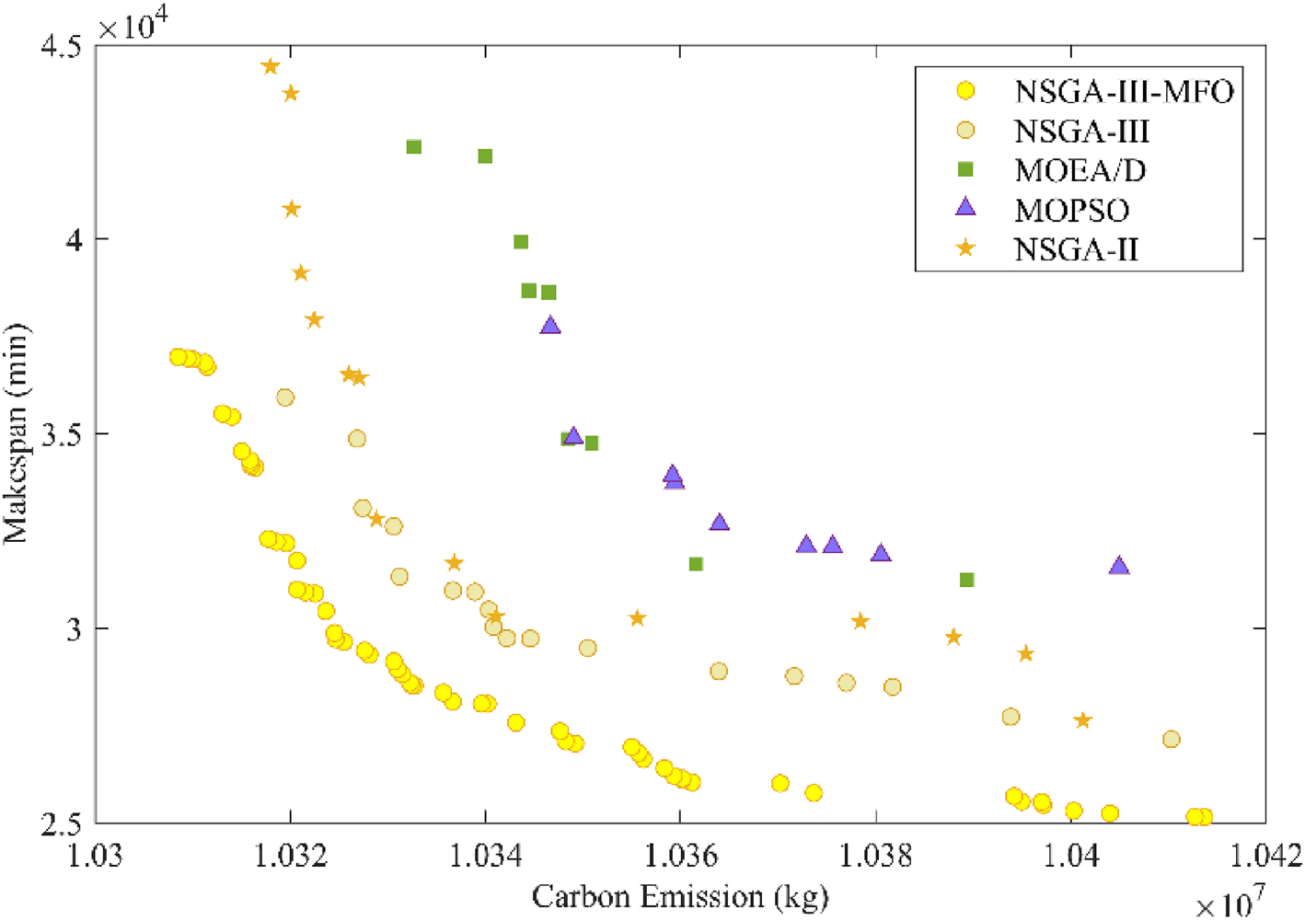
Pareto front of different algorithms.

To validate the performance of the NSGA-III-MFO algorithm, this section compares the optimization results with those of four other algorithms: NSGA-III, NSGA-II, MOEA/D, and MOPSO. The Pareto optimal solutions obtained using different algorithms are shown in Fig. 7. From the figure, we can preliminarily see that the NSGA-III-MFO algorithm has a significant advantage over the other algorithms in solving the problem proposed in this paper.

To further evaluate the performance of the NSGA-III-MFO algorithm, this paper introduces two commonly used evaluation indicators: generational distance (GD) and inverse generational distance (IGD). GD can measure the convergence of the algorithm, while IGD can evaluate the algorithm's extensibility and distribution. The smaller the values of these two indicators, the better the algorithm's performance. The evaluation indicator values for each algorithm are shown in Table 6. The results of Table 6 indicate that the NSGA-III-MFO algorithm has lowest GD and IGD than other algorithms. This means that this NSGA-III-MFO has best performance.

**Table 6:** Evaluation metric values for each algorithm.

	NSGA-III-MFO	NSGA-III	NSGA-II	MOEA/D	MOPSO
GD	0	3418.57	5833.9	10,091.85	7658.77
IGD	0	4453.6	6119.65	14,095.07	18,701.77

#### Experimental results

Current research on crankshaft scheduling mainly focuses on its machining stage. However, the single and double jacketing processes in the actual machining process also consume a significant amount of time and generate a large amount of carbon emissions. The single and double jacketing processes and machining interact with each other, so the collaborative scheduling research of crankshaft machining, single and double jacketing processing helps improve crankshaft production efficiency and reduce carbon emissions.

**Figure 8:**
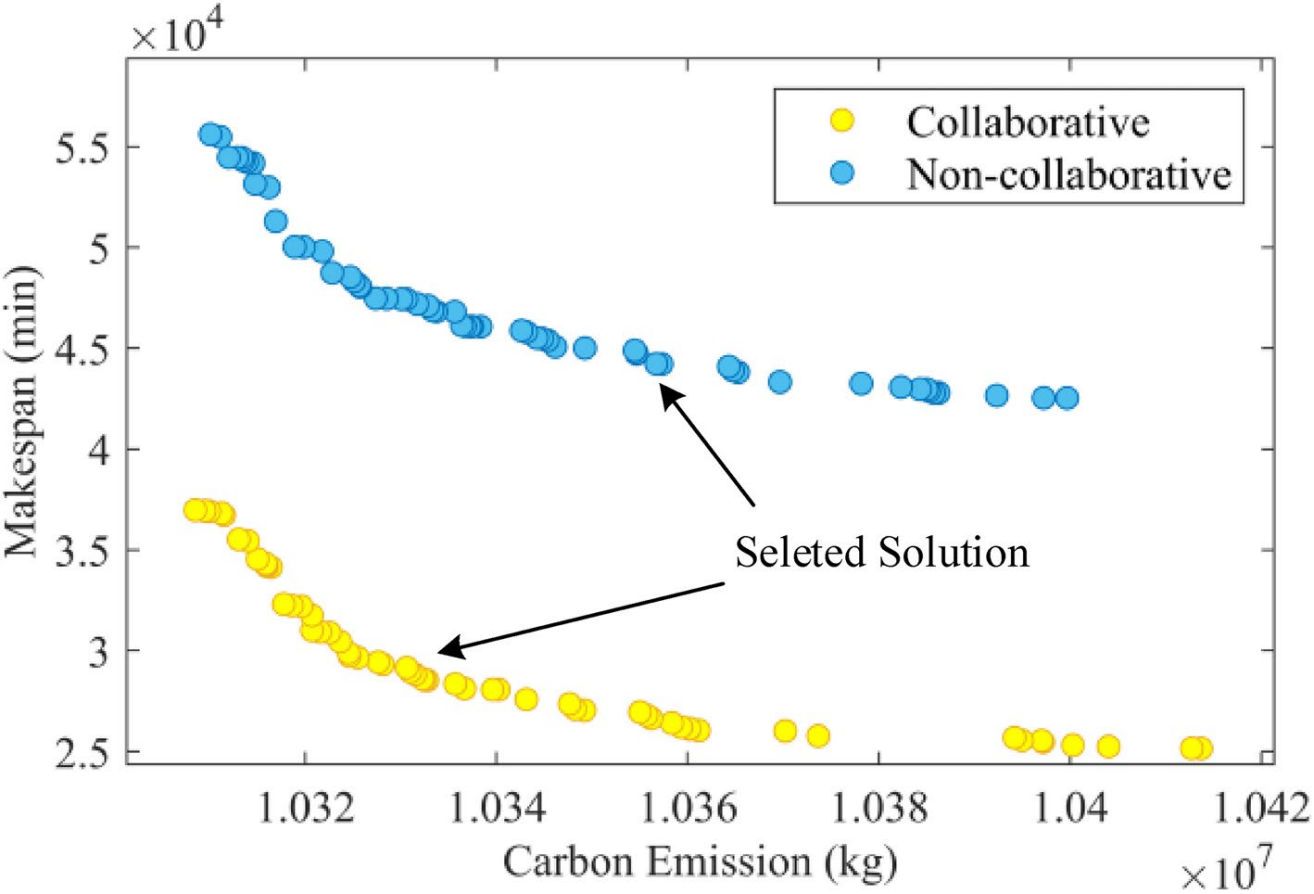
Pareto front of cooperative scheduling and non-cooperative scheduling.

**Figure 9:**
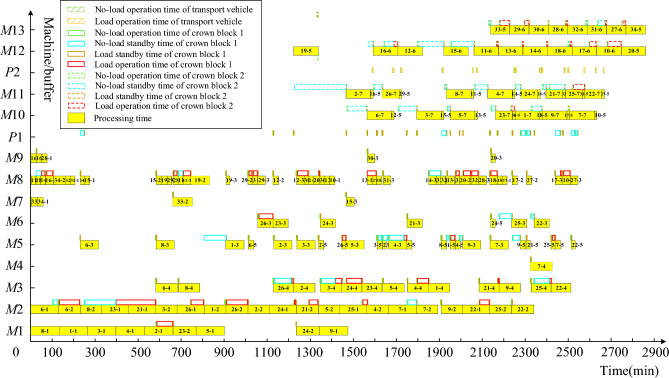
Gantt chart of cooperative scheduling optimization results.

**Figure 10:**
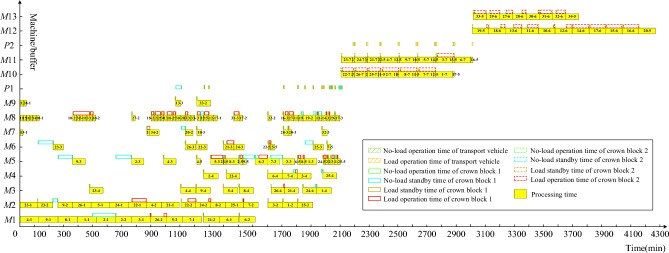
Gantt chart of non-cooperative scheduling optimization results.

For fairness in comparison, both collaborative scheduling and non-collaborative scheduling use the NSGA-III-MFO algorithm and adopt the same parameters. Similarly, to enhance the reliability of the results, the calculations for non-collaborative scheduling are also run 10 times. The final Pareto front is shown in Fig. 8. From Fig. 8, one solution is chosen from the Pareto front solutions of both collaborative and non-collaborative scheduling, and the corresponding Gantt charts are plotted. Figure 9 shows the solution for the collaborative scheduling of crankshaft machining, single and double jacketing processes. Figure 10 presents the solution obtained by optimizing scheduling for each crankshaft machining stage separately. Comparing Figs. 9 and 10, we can see that collaborative scheduling for crankshaft machining contributes to a better overall process, including machining efficiency and carbon emissions. In addition, Table 7 shows the average values of the final Pareto front, where collaborative scheduling is relatively better in terms of maximum manufacturing time and carbon emissions. Carbon emissions are reduced by approximately 3619.74 kg, and the maximum completion time is also reduced by about 17,637.81 min. Thus, the multi-stage collaborative scheduling of the crankshaft manufacturing process can significantly improve machining efficiency and reduce carbon emissions.

**Table 7:** Average values of pareto frontier.

	Maximum completion time (min)	Carbon emissions (kg)
Collaborative scheduling	29,809.01	10,338,440.85
Non-collaborative scheduling	47,446.82	10,342,060.59
GAP	17,637.81	3619.74

## Conclusions

In order to optimize the production efficiency of crankshaft manufacturing enterprises and further promote the green manufacturing of enterprises, this paper proposes a sustainable collaborative scheduling problem for multi-stage mixed flow shop with transportation processes in crankshaft manufacturing. For this problem, a mixed-integer programming model is established, with the minimization of the maximum completion time and carbon emissions as the optimization objectives. An enhanced NSGA-III heuristic optimization algorithm, utilizing the MFO algorithm, is proposed to solve this mathematical model. The case study demonstrates that the integrated optimization algorithm can effectively solve the multi-objective optimization problem studied in this paper. Compared with the classic optimization algorithms for solving multi-objective optimization problems, such as NSGA-III, NSGA-II, MOEA/D, and MOPSO, the NGSA-III-MFO algorithm shows an absolute advantage in optimization results. In addition, this paper compares the multi-stage optimization collaborative scheduling research and non-collaborative scheduling research for crankshaft manufacturing, and the results show that collaborative scheduling research contributes to a more optimal overall realization of the crankshaft manufacturing process, significantly improving production efficiency and reducing carbon emissions. The results indicate that the annual carbon emissions can reduce about 3.6 ton.

While this study provides meaningful insights, it is not without certain limitations. Future research could concentrate on several key areas to address these shortcomings and enhance the robustness and practicality of the findings.

The model does not currently take into account unexpected situations such as equipment failures, which are a common occurrence in manufacturing processes. Future studies could focus on integrating contingency planning and risk mitigation strategies into the model. This would increase its reliability and ensure that it remains effective even in the face of unforeseen disruptions.

Also, the current model could benefit from the design and implementation of more efficient optimization algorithms. While the existing algorithm demonstrates promising results, there is always room for enhancement. Future work could delve into creating and testing new algorithms that further improve efficiency and reduce computational resources. This, in turn, could lead to more sustainable and efficient manufacturing processes in practice. By focusing on these directions, future research can contribute to a more comprehensive and effective approach to sustainable manufacturing.

## Data Availability

The datasets used and/or analysed during the current study available from the corresponding author on reasonable request.
